# MTHFD2 in healthy and cancer cells: Canonical and non-canonical functions

**DOI:** 10.1038/s44324-024-00005-6

**Published:** 2024-03-15

**Authors:** Natalia Pardo-Lorente, Sara Sdelci

**Affiliations:** Centre for Genomic Regulation (CRG), The Barcelona Institute of Science and Technology, Barcelona, Spain

**Keywords:** Cancer metabolism, Cancer, Cell biology

## Abstract

Methylenetetrahydrofolate dehydrogenase 2 (MTHFD2) is a mitochondrial enzyme of the folate-mediated one-carbon metabolism pathway. MTHFD2 has become a highly attractive therapeutic target due to its consistent upregulation in cancer tissues and its major contribution to tumor progression, although it also performs vital functions in proliferating healthy cells. Here, we review the diversity of canonical and non-canonical functions of this key metabolic enzyme under physiological conditions and in carcinogenesis. We provide an overview of its therapeutic potential and describe its regulatory mechanisms. In addition, we discuss the recently described non-canonical functions of MTHFD2 and the mechanistic basis of its oncogenic function. Finally, we speculate on novel therapeutic approaches that take into account subcellular compartmentalization and outline new research directions that would contribute to a better understanding of the fundamental roles of this metabolic enzyme in health and disease.

## One-carbon metabolism

**Fig. 1:**
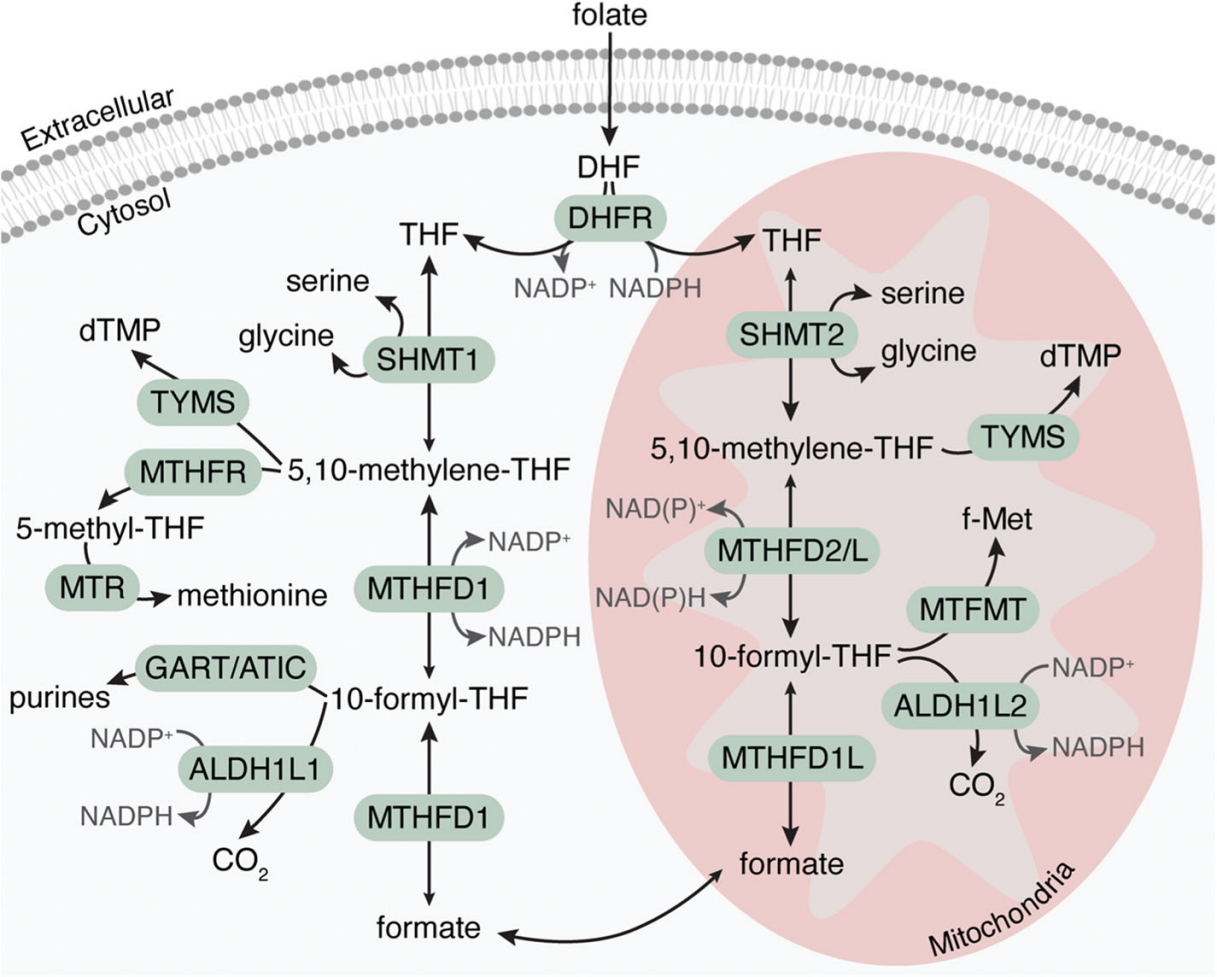
One-carbon metabolism is a compartmentalized pathway primarily localized in the cytosol and mitochondria. ALDH1L1/2, 10-formyltetrahydrofolate dehydrogenase cytosolic (1)/ mitochondrial (2); ATIC, 5-aminoimidazole-4-carboxamide ribonucleotide formyltransferase/IMP cyclohydrolase; DHF, dihydrofolate; DHFR, dihydrofolate reductase; dTMP, deoxythymidine monophopshate; f-Met, formylmethionine; GART, phosphoribosylglycinamide formyltransferase; MTFMT, mitochondrial methionyl-tRNA formyltransferase; MTHFD1, methylenetetrahydrofolate dehydrogenase cyclohydrolase and formyltetrahydrofolate synthase 1; MTHFD1L, monofunctional formyltetrahydrofolate synthase, mitochondrial; MTHFD2/L, methylenetetrahydrofolate dehydrogenase 2/2-like; MTHFR, methylenetetrahydrofolate reductase; MTR, methionine synthase; NAD, nicotinamide adenine dinucleotide; NADP, nicotinamide adenine dinucleotide phosphate; SHMT1/2, serine hydroxymethyltransferase cytosolic(1)/ mitochondrial (2); THF, tetrahydrofolate; TYMS, thymidylate synthase.

One-carbon metabolism is a central pathway in both physiological and pathological contexts. This pathway consists of a complex set of compartmentalized reactions primarily localized in the cytosol and mitochondria (Fig. 1). It is essential for the synthesis of metabolites required for multiple biological processes, such as de novo purine and pyrimidine biosynthesis, amino acid homeostasis, synthesis of S-adenosylmethionine (SAM) required for DNA and histone methylation, and the maintenance of the redox state of the cell^1^. Thus, this pathway regulates the nutritional status of the cell not only through amino acid homeostasis or nucleotide precursor production, but also through the regulation of epigenetic and redox states.

To function as a one-carbon unit carrier, extracellular folate in the cell is first reduced to dihydrofolate (DHF) and then to tetrahydrofolate (THF) by the enzyme dihydrofolate reductase (DHFR)^1^. THF is used in the first step of the folate cycle in the interconversion of serine and glycine. The enzymes serine hydroxymethyltransferase 1 (SHMT1) in the cytosol or serine hydroxymethyltransferase 2 (SHMT2) in the mitochondria catalyze the reversible conversion of serine to glycine by transferring a one-carbon unit from serine to THF to form 5,10-methylene-THF. Subsequently, 5,10-methylene-THF can be reversibly oxidized to 10-formyl-THF by the cytosolic enzyme methylenetetrahydrofolate dehydrogenase 1 (MTHFD1) or the mitochondrial MTHFD2 and MTHFD2-like (MTHFD2L). This reaction is coupled to the oxidoreduction of nicotinamide adenine dinucleotide phosphate (NADP^+^) in the cytosol and NADP^+^ and nicotinamide adenine dinucleotide (NAD^+^) in the mitochondria. To complete the folate cycle, the cytosolic enzyme MTHFD1 or the mitochondrial MTHFD1-like (MTHFD1L) can reversibly hydrolyze 10-formyl-THF to formate, which can then be shuttled between the two subcellular compartments^1,2^. Alternatively, cytosolic or mitochondrial 10-formyl-THF can be eliminated by its complete oxidation to CO_2_ coupled to NADP^+^ reduction by either cytosolic 10-formyltetrahydrofolate dehydrogenase 1-L1 (ALDH1L1) or the mitochondrial form ALDH1L2^3^.

The one-carbon moieties with different oxidation states, 5,10-methylene-THF, 5-methyl-THF and 10-formyl-THF, support distinct biosynthetic functions. This pathway is critical for rapid proliferation and provides the metabolic intermediates required for nucleotide biosynthesis. First, the enzyme thymidylate synthase (TYMS) uses 5,10-methylene-THF as a substrate to convert deoxyuridine monophosphate (dUMP) to deoxythymidine monophosphate (dTMP) in the cytosol and mitochondria, an important step in pyrimidine biosynthesis. Second, the cytosolic pool of 10-formyl-THF is utilized by the enzymes phosphoribosylglycinamide formyltransferase (GART) and 5-aminoimidazole-4-carboxamide ribonucleotide formyltransferase /IMP cyclohydrolase (ATIC) during the de novo purine synthesis pathway^1^.

As mentioned above, the folate pathway can also affect epigenetic maintenance, specifically DNA and histone methylation. In the folate pathway, 5,10-methylene-THF is reduced to 5-methyl-THF by the enzyme methylene tetrahydrofolate reductase (MTHFR), coupled to the oxidation of NADPH. This 5-methyl-THF is then used for the remethylation of homocysteine to methionine by the enzyme methionine synthase (MTR) in the methionine salvage pathway^2^. Methionine is the substrate for the SAM synthetase methionine adenosyltransferases (MAT) enzymes to produce the methylation donor SAM^4^.

One-carbon metabolism also affects translation regulation in the mitochondria, particularly the initiation of translation of mitochondrial-encoded proteins, since mitochondrial 10-formyl-THF can be used by the enzyme methionyl-tRNA formyltransferase (MTFMT) for the formylation of mitochondrial initiator methionine tRNAs^5^. It also contributes to the homeostasis of the amino acids glycine, serine and methionine, and to redox defense, since folate reactions influence both the NAD^+^/NADH and NADP^+^/NADPH ratios in mitochondria and NADP^+^/NADPH in the cytosol^6^. Equally important for redox defense is the synthesis of glycine, which impacts the production of the antioxidant glutathione^1^.

Although most proliferative cells use the mitochondrial one-carbon metabolism pathway to make one-carbon units in the reductive direction, the inactivation of the mitochondrial path through the knock-out of SHMT2, MTHFD2 or MTHFD1L reverses the cytosolic flux so that one-carbon units are produced in the cytosol to ensure the generation of 10-formyl-THF, hence sustaining a rapid growth^7^. This suggests that the folate pools control the direction of the folate pathway flux.

The compartmentalization of the one-carbon folate pathway, with each compartment containing specific enzymes along with their substrates and products, maybe an evolutionary strategy to reduce interference and uncouple one-carbon metabolism from glycolysis^8^. If one-carbon oxidation were to occur in the cytosol, producing NADH, it would disturb the redox balance that controls glucose fermentation to lactate and block glycolysis^1^. Therefore, this subcellular compartmentalization appears to be an evolutionary adaptation to ensure cellular robustness.

## Canonical function of MTHFD2

**Fig. 2:**
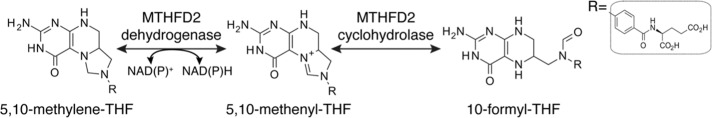
MTHFD2 catalytic activities in the folate pathway. MTHFD2 catalyzes the reversible conversion from 5,10-methylene-tetrahydrofolate (THF) to 5,10-methenyl-THF, which is then converted to 10-formyl-THF, by two sequential dehydrogenase and cyclohydrolase steps. NAD, nicotinamide adenine dinucleotide; NADP, nicotinamide adenine dinucleotide phosphate. Chemical structures were taken from Kawai et al.^56^.

Methylenetetrahydrofolate dehydrogenase 2 (MTHFD2) is a key enzyme in mitochondrial one-carbon metabolism. This bifunctional enzyme catalyzes the reversible conversion of 5,10-methylene-THF to 5,10-methenyl-THF by a dehydrogenase reaction, followed by the conversion of 5,10-methenyl-THF to 10-formyl-THF by a cyclohydrolase reaction (Fig. 2). These reactions can also be catalyzed by the isoenzymes MTHFD1 and MTHFD2-like (MTHFD2L). Continuing the folate cycle, 10-formyl-THF is then converted to formate in the mitochondria by MTHFD1L and translocated to the cytosol, where it is utilized by its cytoplasmic homolog MTHFD1^1^. As mentioned above, mitochondrial 5,10-methylene-THF is used by TYMS for de novo thymidylate synthesis, and mitochondrial 10-formyl-THF is used by MTFMT for the formylation of mitochondrial initiator methionine tRNAs.

The mitochondrial enzyme MTHFD2 differs from its cytosolic counterpart MTHFD1 in that it lacks the synthase domain and has a unique requirement for inorganic phosphate and magnesium ions to support its dehydrogenase activity^9^. Furthermore, while MTHFD1 acts as a housekeeping gene and is ubiquitously expressed in tissues^10^, MTHFD2 is normally expressed in embryonic and transformed cells, but it is absent or weakly expressed in differentiated cells^11,12^.

With regards to the mitochondrial isoenzymes MTHFD2 and MTHFD2L, both exhibit dual redox cofactor specificity so that they can utilize either NAD^+^ or NADP^+^ and contribute to both the NAD^+^/NADH and NADP^+^/NADPH ratios in mitochondria, although MTHFD2 uses NADP^+^ with low efficiency^13^. mRNA levels of both isozymes can be detected in human adult tissues, although MTHFD2 mRNA levels were generally higher. Similarly, both isoenzymes are expressed in cancer cells, although MTHFD2 is highly upregulated in a wide range of tumors, in contrast to MTHFD2L. In addition, MTHFD2L does not respond to growth factor stimulation and does not compensate for MTHFD2 inhibition, further supporting a non-oncogenic role for the MTHFD2L isoenzyme^14^.

## Role of MTHFD2 in healthy cells

MTHFD2 levels are elevated during embryogenesis and in highly proliferative adult tissues such as hematopoietic and mesenchymal stem cells, blood myeloid cells, tonsil lymphoid tissue, intestinal crypts or exocrine pancreas^12,15^. Although the function of MTHFD2 under physiological conditions is not well understood, MTHFD2 activity appears to be important in several highly proliferative processes, such as embryogenesis, hematopoiesis, angiogenesis and the immune response.

MTHFD2 is essential for embryogenesis, where mouse genetic studies showed that embryos with a homozygous deletion of MTHFD2 failed to complete embryonic development. These mutant embryos showed normal body development, but exhibited a liver failure in hematopoiesis, with a significant reduction in nucleated cells^16^. MTHFD2 was also found to sustain self-renewal of mouse embryonic stem cells and aid the reprogramming of induced pluripotent stem cells (iPSC) by ensuring the integrity of the mitochondrial activity and supporting DNA repair^17^. In addition, MTHFD2 has an important function in endothelial cells during atherosclerosis, where it is activated by oxidized phospholipids to support glycine synthesis, thereby promoting angiogenesis^18^.

Furthermore, MTHFD2 appears to contribute to the function of the immune system. Upregulation of mitochondrial one-carbon metabolism has been observed during T-cell activation to support purine and thymidine synthesis for T-cell proliferation and survival^19^. In particular, MTHFD2 has recently been shown to be induced in activated T cells to promote T cell proliferation and release of inflammatory cytokines by providing 10-formyl-THF for de novo purine synthesis. In pathogenic inflammatory conditions, MTHFD2 suppresses the anti-inflammatory T regulatory cells, and its inhibition in vivo reduced the severity of the inflammatory diseases experimental autoimmune encephalomyelitis and delayed-type hypersensitivity^20^.

## The importance of MTHFD2 in cancer

A recent meta-analysis study including 19 cancer types highlighted MTHFD2 as the most consistently upregulated metabolic gene in tumors^12^. While MTHFD2 levels are high in various tumors, its expression is low in the majority of normal adult tissues, with the exception of some highly proliferative tissues^12,15^. Besides, MTHFD2 has been reported to be transcribed but not translated in the normal tissues of testis and thymus^10^. Since MTHFD2 does not perform an exclusive enzymatic reaction within the one-carbon pathway, and its dehydrogenase and cyclohydrolase catalytic activities are shared with other members of the MTHFD family, the mitochondrial MTHFD2L and the cytosolic MTHFD1, it is intriguing why the cancer cell particularly increases the expression of MTHFD2.

Many studies have demonstrated the contribution of MTHFD2 to various aspects of cancer development, such as prognosis, cell proliferation, tumor growth, migration and invasion, or metastasis, in a wide range of cancer types (Table 1).

**Table 1:** MTHFD2 relevance in cancer progression.

Cancer type	Prognosis	Cancer cell proliferation in vitro	Tumor growth in vivo	Migration and invasion	Metastasis in vivo
Bladder	Chen et al.^22^ Liu et al.^21^	Chen et al.^22^		Chen et al.^22^	
Breast	Nilsson et al.^12^ Liu et al.^23^	Selcuklu et al.^33^ Nilsson et al.^12^ Koufaris et al.^34^		Lehtinen et al.^15^	
CNS		Nilsson et al.^12^			
Colon	Asai et al.^57^ Ju et al.^24^	Nilsson et al.^12^ Ju et al.^24^ Wei et al.^35^ Yan et al.^36^	Ju et al.^24^	Wei et al.^35^ Yan et al.^36^	Ju et al.^24^
Esophageal	Wang et al.^25^				
Head and neck	Cui et al.^26^				
Gastric		Tong et al.^37^			
Glioma		Xu et al.^38^		Xu et al.^38^	
Leukemia		Pikman et al.^39^ Gu et al.^40^	Pikman et al.^39^ Gu et al.^40^		
Liver	Liu et al.^27^ Ren et al.^28^			Liu et al.^27^	
Lung	Asai et al.^57^ Nishimura et al.^29^ Shi et al.^30^	Nilsson et al.^12^ Nishimura et al.^29^ Yu et al.^41^ Shi et al.^30^ Gao et al.^42^	Nishimura et al.^29^ Yu et al.^41^ Shi et al.^30^	Shi et al.^30^	Shi et al.^30^
Melanoma		Nilsson et al.^12^			
Ovarian		Nilsson et al.^12^ Li et al.^43^	Li et al.^43^	Li et al.^43^	
Pancreas	Noguchi et al.^31^		Shang et al.^46^		
Prostate		Pällmann et al.^44^	Pällmann et al.^44^		
Renal	Lin et al.^32^	Nilsson et al.^12^ Lin et al.^32^ Green et al.^45^	Green et al.^45^	Lin et al.^32^ Green et al.^45^	

*CNS* central nervous system.

MTHFD2 is elevated in many cancer types and is associated with poor prognosis in several tumors^12,21–32^. A myriad of studies highlight the contribution of MTHFD2 in driving cancer progression. Nilsson and colleagues showed that downregulation of MTHFD2 decreased cell proliferation in a panel of breast, central nervous system, colon, lung, melanoma, ovarian and renal cancer cell lines^12^. In addition, MTHFD2 is known to promote cancer cell proliferation and survival in vitro^22,24,29,30,32–45^ and support tumor growth in vivo^24,29,30,39–41,43–46^ across multiple cancer types. Besides, MTHFD2 supports the metastatic properties of cancer by promoting cell migration and invasion across various cancer cell lines^15,22,27,30,32,35,36,38,43,45^ and metastasis in vivo in colon^24^ and lung^30^ cancer.

Furthermore, MTHFD2 enhances cancer stem-like properties in breast^15^ and lung^29^ cancer. Consistent with this, MTHFD2 suppression has been shown to promote myeloid differentiation in leukemia cell lines^39^. Finally, MTHFD2 may contribute to cancer cell survival through other mechanisms, such as the regulation of the immune response^26,46^ or promotion of drug resistance^29^. Indeed, MTHFD2 downregulation sensitizes breast, liver and kidney cancer cells to the treatment with the chemotherapeutic agent methotrexate^15,27,32^, and increases the sensitivity of radiation-resistant head and neck squamous cell carcinoma cells to the combinatorial treatment with the radiation sensitizer β-lapachone and radiotherapy^47^.

## MTHFD2 as a therapeutic target

Because MTHFD2 is upregulated and drives cancer progression in a wide variety of tumors, and its expression is low in proliferative and differentiated adult tissues^12,15^, this enzyme has become very attractive for the development of novel, metabolically-targeted cancer therapies. In fact, many authors have highlighted the potential of MTHFD2 as a promising therapeutic target in cancer^8,48–51^.

The study of the structure of MTHFD2 has been of great importance for rational drug design^8,9,49^. MTHFD2 forms a dimer, which is achieved by key stabilizing interactions in the interaction of two NAD^+^-binding domains^8,52^, and its crystal structure was reported in 2017 by Gustafsson and colleagues^53^. However, the high degree of structural similarity between the isoenzymes MTHFD2, MTHFD2L and MTHFD1 makes it highly unlikely that competitive inhibitors will achieve enzyme specificity^49^.

**Fig. 3:**
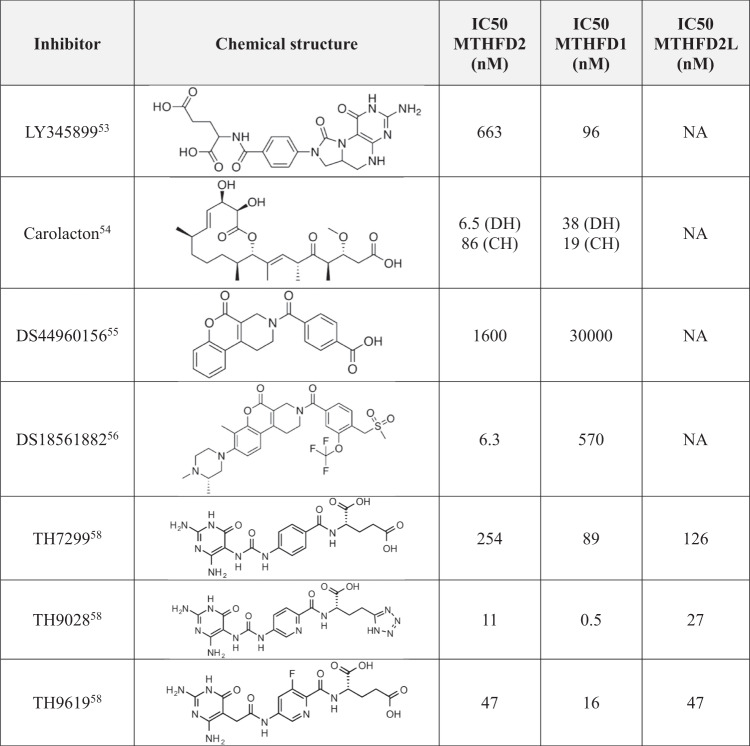
List of MTHFD2 inhibitors. Chemical structures were obtained from Yang et al.^50^ and Bonagas et al.^58^. In all studies, the half maximal inhibitory concentration (IC50) was obtained by biochemical enzymatic assays. CH cyclohydrolase, DH dehydrogenase, NA not available.

Several MTHFD2 inhibitors have already been developed (Fig. 3). The first reported MTHFD2 inhibitor was LY345889^53^, a folate analog that binds within the large cleft between the N-terminal and C-terminal domains of the enzyme, specifically through two hydrogen bonds between Asp155 in MTHFD2 and the pteridine of LY345899. However, selectivity for MTHFD2 was low with half-maximal inhibitory concentration (IC50) values of 663 nM for MTHFD2 and 96 nM for MTHFD1. LY345889 reduced tumor growth in a mouse xenograft model of colon cancer^24^.

Interestingly, the bacterial compound carolacton, a keto-carboxylic acid produced by the myxobacterium *Sorangium cellulosum*, is able to bind to and inhibit MTHFD1 and MTHFD2 enzymes at nanomolar concentrations. This natural product inhibited the growth of endocervical adenocarcinoma and colorectal cancer cells, achieving a maximum inhibition of 80%^54^. Carolacton showed a different selectivity for MTHFD2 and MTHFD1 for the dehydrogenase and cyclohydrolase functions.

Later on, two new MTHFD2 inhibitors were reported, DS44960156^55^ and the optimized version DS18561882, with a much better selectivity for MTHFD2 versus MTHFD1, with an IC50 of 6.3 nM and 570 nM, respectively. The latter inhibitor suppressed tumor proliferation in a murine xenograft model of breast cancer^56^.

In addition, Asai and colleagues^57^ identified several compounds as potential candidates for MTHFD2 inhibition through in silico screening. These candidates bound to MTHFD2 with high specificity and affected the levels of MTHFD2-related metabolites.

Recently, novel MTHFD2 inhibitors TH7299, TH9028 and TH9619 were developed that target MTHFD2 and MTHFD1 at nanomolar concentrations. These inhibitors impaired the proliferation of acute myeloid leukemia cells in vitro and in vivo without significant adverse effects^58^. The anti-cancer effect of the inhibitors was mediated by preventing thymidine production, resulting in the misincorporation of uracil into DNA with subsequent replication stress and accumulation of DNA damage. These MTHFD2 inhibitors were synergistic with dUTPase and ATR serine/threonine kinase inhibitors. Later, these authors reported that TH9619 inhibited cytosolic MTHFD1 and nuclear MTHFD2, but not mitochondrial MTHFD2, because the compound was unable to cross the mitochondrial barrier^59^. Therefore, they showed that the anti-cancer effect of TH9619 is the result of the accumulation of 10-formyl-THF as a consequence of MTHFD1 inhibition while mitochondrial MTHFD2 is still active, causing a folate trap that provokes cell death in breast and colon cancer cells due to thymidylate depletion. This phenomenon is exacerbated by the presence of hypoxanthine under physiological conditions, as it blocks the de novo purine biosynthesis pathway in favor of the salvage pathway, hence preventing the consumption of 10-formyl-THF. The fact that mitochondrial MTHFD2 is required for mediating TH9619 inhibitor’s toxicity in cancer cells explain why these inhibitors do not have adverse effects in normal cells, where MTHFD2 is weak or not expressed.

It is important to note that, although it has been previously shown that inhibition of mitochondrial one-carbon metabolism reverses cytosolic folate metabolism to produce one-carbon units^7^, most of the reported inhibitors inhibit not only MTHFD2 but also cytosolic MTHFD1^53,58^, due to their high structural similarity, thus inhibiting both mitochondrial and cytosolic folate metabolism. Because of the high structural similarity between the MTHFD2, MTHFD2L and MTHFD1 isoenzymes, it is difficult to design compounds with high specificity for MTHFD2. Since MTHFD1 is expressed in normal cells, its inhibition could cause undesired side effects. Additionally, except for the inhibitors TH7299, TH9028 and TH9619, the effect of previous inhibitors on MTHFD2L inhibition has not been studied. To achieve better MTHFD2 selectivity, Tedeschi et al. suggested to take advantage of the single different residue 243 of MTHFD2 and MTHFD2L (Arg243 versus Tyr243)^49^, and Zhao et al. proposed to exploit a novel pocket, distinct from the cofactor and substrate pockets that is formed at the interface between MTHFD2 monomers during dimerization^8^.

Another concern is that, while MTHFD2 depletion appears to inhibit tumor growth, it is not sufficient to kill cancer cells. In fact, MTHFD2 is not a highly essential gene in cancer cell lines, according to the DepMap portal^60^. For this reason, CRISPR knock-out screenings could help to elucidate MTHFD2 synthetic lethalities as candidates for combinatorial treatments. In this regard, we recently performed a whole-genome CRISPR knock-out screening that revealed a synthetic lethality between MTHFD2 and topoisomerase 2A (TOP2A)^61^. TOP2A is required for mitotic chromosome condensation^62^ and DNA replication and repair^63^, and since the topoisomerase 2 inhibitor etoposide is widely used in clinics as a chemotherapeutic agent in combinatorial therapies in lung cancer^64^, breast cancer^65^, prostate cancer^66^ and lymphoma^67^, among other cancer types, the combinatorial inhibition of MTHFD2 and topoisomerase 2 could become a potential cancer therapeutic approach.

In addition, MTHFD2 silencing has previously been shown to increase the sensitivity of breast cancer cells to glycine and folate depletion^34^ and the sensitivity of leukemia cells to glycine depletion^39^. Similarly, inhibition of the mitochondrial one-carbon metabolism rendered cancer cells dependent on extracellular serine and glycine^7^. Further research in this area is needed to explore the possibility of combining MTHFD2 inhibition with dietary interventions to improve cancer cell death.

## Regulation of MTHFD2 in cancer and healthy cells

**Fig. 4:**
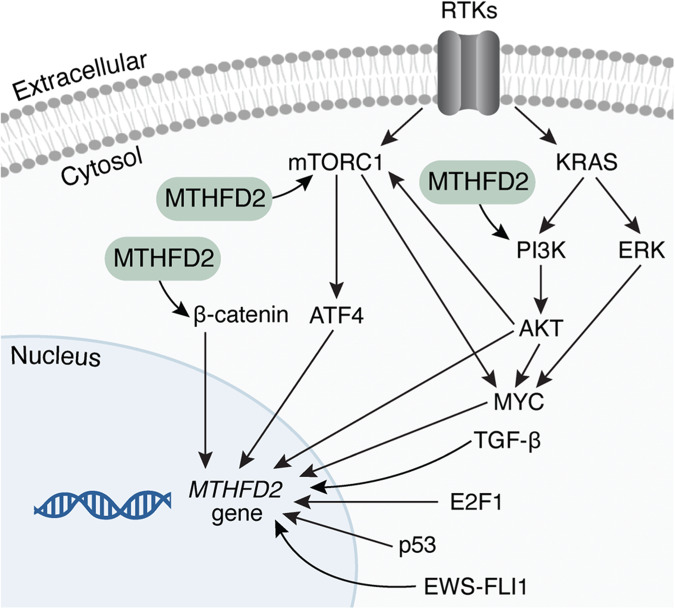
Transcriptional regulation of MTHFD2. MTHFD2 upregulation can be promoted by several signaling pathways. Besides, MTHFD2 also induces the activation of some of these signaling pathways. AKT protein kinase B, ATF4 activating transcription factor 4, ERK extracellular signal-regulated kinase, HIF2A hypoxia inducible factor 2α, mTORC1 mammalian target of rapamycin complex 1, PI3K phosphatidylinositol 3-kinase, RTKs receptor tyrosine kinases, TGF-β transforming growth factor β.

MTHFD2 is rarely mutated or amplified in cancer^68^, supporting that its elevated levels in cancer are a consequence of its transcriptional upregulation. Several studies have described the potential regulatory mechanisms underlying MTHFD2 expression (Fig. 4), including transcriptional, post-transcriptional and post-translational regulation.

Studies in murine fibroblasts have shown that mammalian target of rapamycin complex 1 (mTORC1) signaling induces MTHFD2 expression through the activation of activating transcription factor 4 (ATF4), thereby stimulating purine biosynthesis^69^. On the other hand, MTHFD2 can also regulate mTORC1 activity in T cells. Sugiura et al. observed that MTHFD2 downregulation resulted in the accumulation of the metabolic intermediate 5-aminoimidazole-4-carboxamide-1-beta-D-ribofuranosyl 5’-monophosphate (AICAR), a regulator of the mTORC1/AMP-activated protein kinase (AMPK) signaling pathway, and its inhibition decreased the phosphorylation of mTORC1 targets^20^.

In prostate cancer cells, ATF4 was found to induce the expression of several mitochondrial one-carbon metabolism enzymes, including not only MTHFD2 but also MTHFD1L and SHMT2^44^. The authors showed that mitochondrial one-carbon metabolism is differentially regulated in prostate cancer compared to normal prostate, with mTORC1 signaling supporting its upregulation in normal tissues and MYC in cancerous tissues. Similarly, in lung cancer, ATF4 has been shown to influence MTHFD2 expression through MYC upregulation^42^. In leukemia, the MYC oncogene has also been highlighted as a potential upstream regulator of MTHFD2, since MYC downregulation decreased the expression of MTHFD2 and other mitochondrial one-carbon metabolism enzymes, such as MTHFD1L and SHMT2, and was found to bind to the promoter region of these folate genes in Burkitt lymphoma, multiple myeloma, chronic myeloid leukemia, glioblastoma multiforme and small cell lung cancer cell lines^39^.

Additionally, in colorectal cancer cells, MYC transcriptionally upregulated MTHFD2 through the activation of KRAS and its downstream signaling pathways protein kinase B (AKT) and extracellular signal-regulated kinase (ERK) 1/2^24^, as the activation of KRAS/AKT/ERK pathways can stabilize MYC by inhibiting its ubiquitin-mediated degradation^70^. In lung cancer, Moran and colleagues also reported an association between KRAS and MTHFD2 expression^71^. MTHFD2 can also be induced by interferon-γ via> the AKT-mTORC1 pathway in several cancer types, resulting in the upregulation of programmed death-ligand 1 (PD-L1) and immune evasion. Again, the MTHFD2-induced upregulation of PD-L1 was mediated by increasing MYC stability through the production of sufficient uridine-related metabolites for MYC O-GlcNAcylation^46^.

Other studies suggest that the regulation between AKT/MYC signaling networks and MTHFD2 may be bidirectional. In bladder cancer cells, MTHFD2 was shown to promote proliferation and migration by upregulating MYC through the AKT pathway^22^. Consistent with these findings, the expression profile driven by MTHFD2 downregulation in bladder cancer cells showed an enrichment of the phosphatidylinositol 3-kinase (PI3K)/AKT pathway and decreased the protein levels of phospho-PI3K/PI3K and phospho-AKT/AKT, demonstrating that MTHFD2 depletion suppresses the PI3K/AKT pathway^72^. Similarly, in lung adenocarcinoma, MTHFD2 promoted tumor progression by activating the AKT/glycogen synthase kinase-3β (GSK3B)/β-catenin signaling cascade^30^. Conversely, MTHFD2 can also be activated by β-catenin in hepatocellular carcinoma, where MTHFD2 was found to mediate the oncogenic function of nucleoside diphosphate kinase 7 (NME7), which induces the Wnt/β-catenin pathway that activates MTHFD2 expression^28^.

In ovarian cancer cells, MTHFD2 promoted cancer cell progression by activating the signal transducer and activator of transcription 3 (STAT3) pathway, which has been implicated in cancer cell proliferation and metastasis^43^. In colon and osteosarcoma cell lines, MTHFD2 has been identified as a transcriptional target of p53, although its depletion can also activate p53 and its target p21 via AMPK signaling stimulation caused by the accumulation of the metabolic intermediate AICAR^73^. Other studies have shown that, in breast cancer, transforming growth factor-β (TGF-β) can induce the expression of MTHFD2^15^; in bladder cancer cells, MTHFD2 expression is regulated by the transcription factor E2F1^21^; in kidney cancer, MTHFD2 was found to be a transcriptional target of hypoxia inducible factor 2α (HIF2A)^45^; and in Ewing sarcoma, the chimeric transcription factor EWS-FLI1 drove the expression of MTHFD2 and MTHFD1L^74^.

MTHFD2 expression can also be regulated post-transcriptionally by the expression of microRNAs in acute myeloid leukemia^40^, breast cancer^33,75^, colorectal cancer^36^, gastric cancer^37^ and glioma cells^38^. Furthermore, other studies suggest that MTHFD2 expression may be regulated post-translationally. Acetylation of an active site residue (Lys88) of MTHFD2 can be removed by the NAD-dependent protein deacetylase sirtuin 3 (SIRT3), thereby activating its enzymatic activity^76^. Besides, phosphoproteomic studies have identified five phosphorylation sites on MTHFD2 (S149, T187, T191, T306 and T324)^48^.

Finally, among the mitochondrial one-carbon folate enzymes, MTHFD2 was particularly responsive to mitogenic stimuli in several cancer cells, and its expression was repressed by growth signal withdrawal and rapidly reinduced upon restimulation^77^.

In summary, MTHFD2 upregulation can be driven by various transcriptional, including mTORC1, MYC or Wnt/β-catenin signaling, post-transcriptional and post-translational mechanisms, although the mechanistic basis of this regulation appears to be cancer type specific. An open question is whether the regulatory signaling mechanisms behind the upregulation of MTHFD2 in cancer are shared during embryonic development or whether these regulatory mechanisms are context specific. The results obtained by Pällmann and colleagues support the latter hypothesis, since they showed that mitochondrial one-carbon metabolism is upregulated by MYC in prostate cancer and by mTORC1 in normal prostate^44^. However, further studies in a broader range of tissues are needed to clarify the regulatory mechanisms promoting MTHFD2 upregulation.

## Non-canonical nuclear functions of MTHFD2

There is increasing evidence that enzymes of central metabolism can unexpectedly translocate to the nucleus and affect epigenetics, transcriptional regulation and chromatin remodeling^2,78–81^. The revolutionary concept of compartmentalized metabolism, which is based on on-demand localized production of metabolites to meet specific cellular needs, is replacing the classical concept of central metabolism. The production of nuclear acetyl-CoA via the nuclear translocation of the cytosolic or mitochondrial enzymes acetyl-CoA synthetase 2 (ACSS2)^82^, ATP citrate lyase (ACLY)^83,84^, pyruvate dehydrogenase complex (PDC)^84^ and carnitine acetyltransferase (CAT)^85^; and the synthesis of nuclear SAM upon nuclear translocation of the cytosolic MAT enzymes^86,87^, are key examples of how nuclear metabolism can directly influence epigenetics. The tricarboxylic acid (TCA) cycle is also active in the nucleus, impacting epigenetics, chromatin dynamics and transcription regulation^88,89^. For instance, the nuclear translocation of the TCA enzymes aconitase 2 (ACO2), isocitrate dehydrogenase (IDH1, IDH3G) or oxoglutarate dehydrogenase (OGDH) can promote pluripotency through histone acetylation^82^. In addition, nuclear metabolism can also influence chromatin remodeling by the nuclear translocation of the ADP-sugar pyrophosphatase nudix hydrolase 5 (NUDT5) for nuclear ATP synthesis^90^, or transcriptional regulation by the nuclear production of nucleotides upon nuclear translocation of the cytosolic folate enzyme MTHFD1^91^.

The presence of one-carbon metabolism in the nuclear compartment has also long been recognized, where the folate enzymes DHFR, SHMT1, SHMT2, TYMS and MTHFD1 have previously been shown to be present in the nucleus to support DNA replication and transcription^2,91–94^. MTHFD2 was first reported in 2015 to be present in the nucleus in both cancer cell lines and human tumors, and to be associated with regions of newly replicated DNA, pointing to a role in driving cancer cell proliferation^77^. The authors also suggested that MTHFD2 may drive cell cycle progression, since it was co-expressed with nuclear genes involved in cell cycle regulation, although they did not test this hypothesis.

To date, several non-canonical functions for MTHFD2 have been described. Co-expression and nuclear interactome studies suggest a role for nuclear MTHFD2 in RNA translation and metabolism^95^. MTHFD2 may also influence cancer epitranscriptomics, as it has been shown to promote N6-methyladenosine (m6A) methylation of HIF2A mRNA to enhance its translation in clear cell renal carcinoma^45^. MTHFD2 and HIF2A appear to form a positive feedback loop in kidney cancer, where MTHFD2 stabilizes HIF2A but is also a transcriptional target of HIF2A, as HIF2A promotes MTHFD2 expression by binding to its promoter. In addition, by stabilizing HIF2A, MTHFD2 promotes aerobic glycolysis, linking RNA methylation to the metabolic rewiring that drives tumor growth in renal cell carcinoma^45^. However, Green and colleagues do not explicitly mention whether this epitranscriptomic function is specifically exerted by nuclear MTHFD2.

Furthermore, we showed that MTHFD2, together with other members of the one-carbon pathway, localizes to chromatin in leukemia cells^91^. Similarly, in p53-mutant colon cancer cells, MTHFD2 was detected not only in the nucleus but also in the chromatin fraction, where it is involved in the DNA damage repair response. Specifically, MTHFD2 interacted with the N-terminal domain of poly-ADP-ribose polymerase 3 (PARP3) in the nucleus and enhanced its activity to induce non-homologous end joining (NHEJ) repair in p53-deficient but not wild-type cells^73^. Thus, MTHFD2 prevented DNA damage in p53-mutant cancer cells, and this non-canonical role was independent of its enzymatic activity.

The nuclear localization of MTHFD2 was also confirmed in bladder cancer cells, where nuclear MTHFD2 was shown to interact with cyclin-dependent kinase 2 (CDK2) in bladder cancer tissues and cells, regardless of its phosphorylation status^21^. Here, MTHFD2 induced CDK2-mediated Retinoblastoma (Rb) phosphorylation and activation, which in turn increased E2F1 transcriptional activity, a key regulator of cell cycle phase transition. These authors proposed that the nuclear translocation of MTHFD2 might be related to mitochondrial morphological changes, since they observed that nuclear MTHFD2 was increased in cells synchronized in S phase, and also upon treatment with a fission inhibitor that induces mitochondrial fusion, mimicking S-phase mitochondria.

Recently, we validated the nuclear localization of MTHFD2 in a panel of breast, lung and colon cancer cells, as well as in colon cancer patient-derived organoids^61^. We found that MTHFD2 localizes to the nucleus of cancer cells to maintain DNA and histone methylation and guarantee proper mitotic progression. Genetic or pharmacological disruption of this mechanism resulted in aberrant chromosome congression and segregation during mitosis, which can threaten cancer cell survival. Furthermore, we observed much higher nuclear levels of MTHFD2 in healthy proliferating colon organoids compared to cancer organoids^61^, and we speculate that it may be necessary to ensure accurate mitotic progression and prevent genomic instability.

The recent evidence linking the MTHFD2 nuclear pool and its “moonlighting” functions in DNA replication, RNA metabolism, methylation and DNA damage repair to tumorigenesis render this enzyme an intriguing cancer target. Reasonably, MTHFD2 may fulfill different compartmentalized metabolic demands required for specific biological processes, for instance, by providing metabolites for the de novo pyrimidine synthesis or for methylation reactions, which is crucial to ensure a rapid DNA replication and repair, and for epigenetic maintenance.

## Mechanisms of MTHFD2 oncogenic activity

**Fig. 5:**
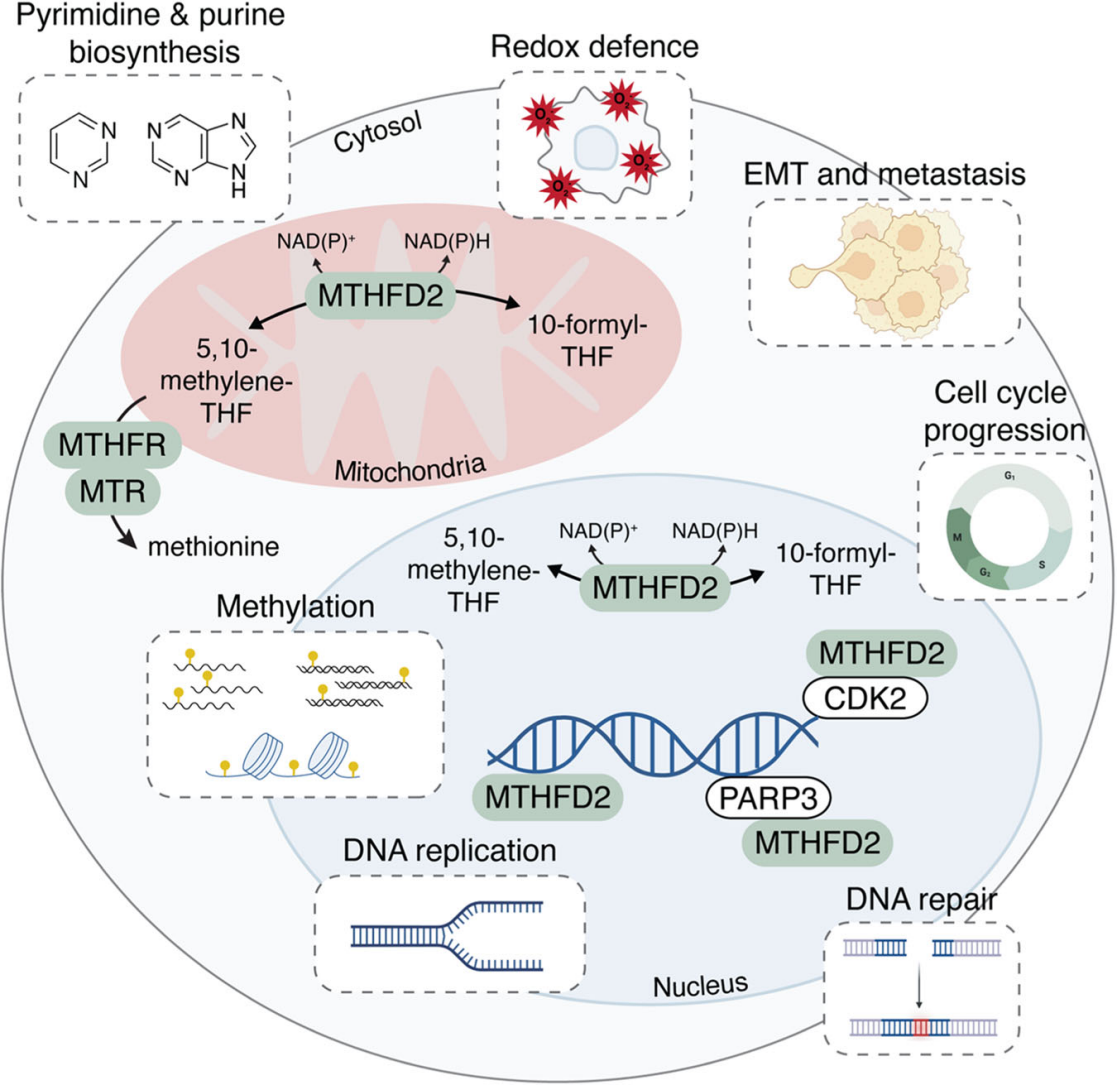
Mechanisms by which MTHFD2 promotes cancer progression. CDK2 cyclin-dependent kinase 2, EMT epithelial-mesenchymal transition, MTHFD2 methylenetetrahydrofolate dehydrogenase 2/2-like, MTHFR methylenetetrahydrofolate reductase, MTR methionine synthase, NAD nicotinamide adenine dinucleotide, NADP nicotinamide adenine dinucleotide phosphate, PARP3 poly-ADP-ribose polymerase 3, THF tetrahydrofolate.

Although the involvement of MTHFD2 in cancer cell proliferation is clear, there are a myriad of mechanistic pathways by which MTHFD2 may contribute to carcinogenesis (Fig. 5).

The canonical mitochondrial function of MTHFD2 could support tumor progression by providing substrates for the de novo synthesis of nucleotide precursors needed for the rapid DNA replication required by cancer cells. In lung cancer cells, MTHFD2 knockdown decreased purine nucleotides, along with an accumulation of the purine intermediate AICAR. Supplementation with the purine derivative hypoxanthine or the folate metabolites 10-formyl-THF and formate partially restored the growth defect found in MTHFD2 downregulated cells, supporting that MTHFD2 induces cell growth by providing substrates for purine biosynthesis^29^. Consistent with this work, MTHFD2 has been shown to promote purine biosynthesis via the mTORC1 signaling pathway upon growth stimulation^69^. On the other hand, in osteosarcoma cells, supplementation with nucleosides, folic acid or thymidine alone was able to rescue cancer cell viability, while supplementation with glycine or hypoxanthine was not, supporting that the antiproliferative effect of MTHFD2 downregulation may be caused by the absence of thymidine^58^. Bonagas and colleagues showed that MTHFD2 prevents replication stress in cancer because MTHFD2 downregulation led to thymidine depletion, which resulted in uracil misincorporation with the subsequent DNA damage at replication forks and impairment of DNA duplication^58^. Interestingly, since MTHFD2 has been shown to colocalize with regions of newly replicated DNA^77^ and to promote DNA damage repair^73^, the compartmentalized supply of metabolites for de novo nucleotide synthesis might be required to promote rapid proliferation. However, these authors observed that proliferation and DNA repair were independent of MTHFD2 catalytic activity, which would contradict this hypothesis.

In addition, MTHFD2 may promote tumor progression by protecting cancer cells from oxidative stress, since MTHFD2 is a NADP^+^/NAD^+^-dependent enzyme that regulates the NADP^+^/NADPH and NAD^+^/NADH ratios. Depletion of both MTHFD1 and MTHFD2 isoenzymes decreased cellular NADPH/NADP^+^ and reduced/oxidized glutathione (GSH/GSSG) ratios, resulting in increased cell susceptibility to oxidative stress^6^. In colon cancer cells, MTHFD2 downregulation also decreased cellular NADPH/NADP^+^ and GSH/GSSG ratios, disrupted redox homeostasis and increased cell toxicity upon hydrogen peroxide addition^24^. The same authors showed that MTHFD2 is required for cell survival during hypoxia, as MTHFD2 downregulation resulted in impaired hypoxic induction of the GSH/GSSG ratio, with a corresponding increase in reactive oxygen species (ROS) levels and cell death. Similarly, MTHFD2 knock-out colon cancer cells showed decreased NADPH and GSH levels and increased ROS levels upon cisplatin treatment^76^. The involvement of MTHFD2 in redox homeostasis was also confirmed in lung cancer cells^42^, where MTHFD2 depletion increased the oxidative damage and decreased the levels of antioxidative factors. Besides, MTHFD2 increased NADH production in both normal and cancer cells when respiration was impaired, either by oxygen deprivation or by inhibition of electron transport chain activity^96^.

The product of MTHFD2, 5,10-methylenetetrahydrofolate, can be converted to SAM by two sequential catalytic reactions carried out by the folate enzymes MTHFR and MTR ^1^. The first reported link between MTHFD2 and epigenetics was the discovery that MTHFD2 can promote the methylation of HIF2A mRNA in renal cancer cells to promote aerobic glycolysis^45^. Notably, Green and colleagues observed a reduction in SAM levels upon MTHFD2 downregulation, supporting the requirement of MTHFD2 for epigenetic maintenance. In line with these findings, we observed that MTHFD2 loss results in profound defects in global DNA methylation and histone methylation at centromeres^61^, reinforcing the key role of MTHFD2 in epigenetic maintenance. Conversely, the levels of the histone methylation marks H3K4me3 and H3K27me3 were increased upon MTHFD2 inhibition in mouse T helper 17 cells but not in T helper 1 and T regulatory cells^20^. This may indicate that the effect of MTHFD2 on epigenetic modifications may be different in cancer cells and immune cells, or that the increased methylation observed in this particular T cell subtype is an indirect rather than a direct consequence of MTHFD2 inhibition.

Other studies suggest that MTHFD2 might drive tumor development by regulating the cell cycle. MTHFD2 downregulation resulted in G0/G1-S delay in colon cancer cells, while its overexpression increased the proportion of cells in S and M phases^35^. In lung cancer, MTHFD2 knockdown decreased the levels of several cell cycle genes, including cyclin-A2 (CCNA2), minichromosome maintenance complex component 7 (MCM7) and S-phase kinase-associated protein 2 (SKP2), in cell lines and tumor xenograft tissues, while the opposite was observed with MTHFD2 overexpression^41^. Similarly, MTHFD2 was positively correlated with the cell cycle pathway in bladder cancer patients, specifically co-expressed with CCNA2 and G2/mitotic-specific cyclin-B1 (CCNB1) genes in bladder tissues^21^. These results show that MTHFD2 can affect cell cycle regulation, but do not clarify whether this regulation is direct or indirect. Liu et al. demonstrated a direct control of the cell cycle by MTHFD2, where nuclear MTHFD2 was shown to interact with CDK2 to promote cell cycle progression in bladder cancer^21^. Additionally, we have also shown the importance of MTHFD2 in ensuring proper mitotic progression, where the absence of MTHFD2 or the catalytic inhibition of nuclear MTHFD2 resulted in several congression and segregation defects during mitosis^61^.

Regarding its metastatic potential, MTHFD2 appears to promote the epithelial-mesenchymal transition by upregulating vimentin and N-cadherin, where MTHFD2 depletion has been shown to impair the organization of the vimentin network required for cell motility and migration in breast cancer cells^15^ and in renal cancer^32^. Besides, cancer cells also overproduce formate beyond its anabolic requirements^97^, and formate has been shown to promote invasiveness and metastasis though lipid reprogramming^97^. Since MTHFD2 operates in the oxidative direction in the mitochondria in cancer cells^1^, producing 10-formyl-THF that can be then converted into formate by mitochondrial MTHFD1L, this could be another mechanism through which MTHFD2 can promote metastasis.

Studying the metabolic rewiring upon MTHFD2 depletion might provide a better understanding of the metabolic contribution of MTHFD2 in tumorigenesis. Suppression of MTHFD2 in breast cancer cells increased the flux of glycolysis and glutamine consumption, as well as their sensitivity to glycine and folate depletion^34^. This increased dependence on glycine upon MTHFD2 suppression was also shown in leukemia cells, accompanied by a depletion of tricarboxylic acid cycle intermediates, an increase in the succinate to α-ketoglutarate ratio and sphingomyelin and triglyceride production^39^. In contrast to Koufaris et al., decreased glycolysis was observed upon MTHFD2 downregulation in renal cell carcinoma cells^45^. Of note, formate supplementation did not rescue cell viability in breast cancer^34^ or leukemia^39^, but did rescue cell proliferation in renal cancer^45^. These evidence suggest that the metabolic reprogramming driven by MTHFD2 may be tissue-dependent.

Finally, there is conflicting evidence regarding the requirement of its catalytic activity for tumorigenesis. While MTHFD2 was found to promote cancer cell proliferation in colon cancer cell lines^77^ and bladder cancer cells^21^, and to prevent DNA damage in p53 mutant cancer cells^73^, regardless of its dehydrogenase activity, its enzymatic activity was required for cancer cell survival in osteosarcoma cells^58^, to confer stem-like properties in lung cancer cells^29^ and to ensure epigenetic maintenance and accurate mitotic progression in colon cancer cells^61^. Nevertheless, the fact that several MTHFD2 inhibitors that inhibit MTHFD2 catalytic activity have shown promising results in impairing cancer progression in vitro and in vivo^53,56,58^ supports that MTHFD2 catalytic function is highly relevant for tumorigenesis. Further research is needed to investigate whether MTHFD2 inhibitors may also affect other functions of the enzyme beyond blocking its catalytic activity, for example, by interfering with MTHFD2’s ability to interact with key partners to perform a non-enzymatic function.

Interestingly, since we know that MTHFD2 can favor cancer progression through various mechanisms, such as providing metabolites for nucleotide synthesis, maintaining epigenetic homeostasis or promoting cell cycle progression, further research should address whether the combination of MTHFD2 inhibition with the inhibition of some of these mechanisms could synergistically boost cancer cell death. For instance, the combined inhibition of MTHFD2 and bifunctional phosphoribosylaminoimidazole carboxylase/phosphoribosylaminoimidazole succinocarboxamide synthetase (PAICS) in purine synthesis has shown promising results in *MYCN*-amplified neuroblastoma^98^.

## Future perspectives

The one-carbon metabolic enzyme MTHFD2 has emerged as the most upregulated metabolic enzyme across cancer, highlighting its potential as a promising cancer therapeutic target. Extensive research has demonstrated the diverse functions by which MTHFD2 promotes cancer progression, including cancer cell proliferation, tumor growth, invasion and metastasis, stem cell-like behavior or drug resistance. Mechanistically, MTHFD2 may promote cancer cell growth by providing precursors for purine and thymidylate biosynthesis, protecting cancer cells from the oxidative stress, sustaining epigenetic maintenance, regulating cell cycle progression and promoting the epithelial-mesenchymal transition, among others. The discovery that MTHFD2 can also be localized in the nucleus of cancer cells in various tumor types opened up a myriad of possibilities by which nuclear MTHFD2 could contribute to cancer progression through non-canonical functions. Some studies have uncovered non-canonical functions of MTHFD2, with nuclear MTHFD2 appearing to be relevant for DNA replication, RNA translation and metabolism, DNA damage repair, and cell cycle or mitotic progression.

Clarifying whether the nuclear pool of MTHFD2 has a key function in cancer progression will shed light on the importance of metabolic subcellular compartmentalization in cancer and better delineate future cancer therapeutic approaches. For example, MTHFD2 inhibitors would be beneficial in a scenario where the nuclear and the mitochondrial pools of MTHFD2 equally promote cancer progression. However, if one or the other pool is found to be much more crucial for tumorigenesis, the usage of compartment-specific degradation tags would be more advantageous^2^. To design more efficient therapeutic interventions, nuclear- and mitochondrial-restricted MTHFD2 cell lines are of prime importance to assess their specific function in tumorigenesis.

Nuclear levels of MTHFD2 were highly abundant in healthy proliferating colon organoids^61^. Consequently, it would be very interesting to further investigate the subcellular compartmentalization of MTHFD2 during embryogenesis and in proliferating adult cells, and whether nuclear MTHFD2 could also play a role in regulating centromere methylation and proper mitotic progression during embryogenesis and/or in proliferating adult tissues, or whether this is a specific role associated with cancer. This is of particular relevance since an accurate mitotic division is essential in embryogenesis to ensure a live birth, but also in proliferative adult tissues to maintain genomic stability. In this context, it would be interesting to explore whether MTHFD2 in the nucleus is also involved in the maintenance of telomeres, which determine the proliferative capacity of the cell.

In addition, the study of redox balance from a compartmentalized point of view has been poorly addressed. MTHFD2 normally functions as a NAD^+^/NADP^+^-consuming enzyme, although it can also function in the opposite direction. Redox balance is known to affect key cellular processes such as cell cycle and mitotic progression^99–101^. Redox homeostasis may also influence other relevant processes such as epigenetic remodeling or DNA damage. For instance, sirtuins are NAD^+^-dependent histone deacetylases, with key functions in chromatin organization, transcriptional regulation and protection against oxidative stress^102^. Similarly, poly-ADP-ribose polymerases (PARPs) are NAD^+^-dependent enzymes that are required not only for DNA damage repair but also for chromatin remodeling^103,104^. Therefore, if MTHFD2 alters the redox balance in the mitochondrial and/or nuclear compartments, this could affect the functionality of NAD^+^-dependent enzymes and, in turn, influence many cellular functions. A further examination about how MTHFD2 activity may affect redox homeostasis activity in different subcellular compartments would provide interesting insights into how redox compartmentalization affects cellular homeostasis.

## Data Availability

No datasets were generated or analysed during the current study.
